# Ephrin-A1 inhibits NSCLC tumor growth via induction of *Cdx-2* a tumor suppressor gene

**DOI:** 10.1186/1471-2407-12-309

**Published:** 2012-07-23

**Authors:** Bhagyalaxmi Sukka-Ganesh, Kamal A Mohammed, Frederic Kaye, Eugene P Goldberg, Najmunnisa Nasreen

**Affiliations:** 1 Division of Pulmonary & Critical Care Medicine, Department of Medicine, University of Florida, Gainesville, FL, USA; 2 NF/SG Veterans Health System, Malcom Randall Veterans Affairs Medical Center, University of Florida, Gainesville, FL, USA; 3 Biomaterial Science and Engineering, University of Florida, Gainesville, FL, USA; 4 Division of Hematology/Oncology, University of Florida, Gainesville, FL, USA

**Keywords:** Receptor EphA2, Ephrin-A1, Claudin-2, *cdx-2*, NSCLC

## Abstract

**Background:**

Tumor formation is a complex process which involves constitutive activation of oncogenes and suppression of tumor suppressor genes. Receptor EphA2 and its ligand ephrin-A1 form an important cell communication system with its functional role in cell-cell interaction and tumor growth. Loss of cell-cell adhesion is central to the cellular transformation and acquisition of metastatic potential. Claudins, the integrated tight junction (TJ) cell-cell adhesion proteins located on the apico-lateral portion of epithelial cells, functions in maintaining cell polarity. There is extensive evidence implicating Eph receptors and ephrins in malignancy, but the mechanisms how these molecular players affect TJ proteins and regulate tumor growth are not clear. In the present study we hypothesized that EphA2 signaling modulates claudin-2 gene expression via induction of *cdx-2*, a tumor suppressor gene in NSCLC cells.

**Methods:**

The expression of EphA2, claudin-2 was determined in various NSCLC cell lines by using real-time quantitative polymerase chain reaction and Western blot analysis. The claudin-2 expression was also analyzed by immunofluorescence analysis. EphA2 and *erk1/erk2* phosphorylation in ephrin-A1 activated cells was evaluated by Western blot analysis. The cell proliferation and tumor colony formation were determined by WST-1 and 3-D matrigel assays respectively.

**Results:**

NSCLC cells over expressed receptor EphA2 and claudin-2. Ephrin-A1 treatment significantly down regulated the claudin-2 and EphA2 expression in NSCLC cells. The transient transfection of cells with vector containing ephrin-A1 construct (pcDNA-EFNA1) decreased the expression of claudin-2, EphA2 when compared to empty vector. In addition ephrin-A1 activation increased c*dx-2* expression in A549 cells. In contrast over-expression of EphA2 with plasmid pcDNA-EphA2 up regulated claudin-2 mRNA expression and decreased *cdx-2* expression. The transient transfection of cells with vector containing *cdx-2* construct (pcMV-*cdx-2*) decreased the expression of claudin-2 in A549 cells. Moreover, silencing the expression of receptor EphA2 by siRNA significantly reduced claudin-2 expression and decreased cell proliferation and tumor formation. Furthermore, silencing *cdx-2* gene expression before ephrin-A1 treatment increased claudin-2 expression along with increased cell proliferation and tumor growth in A549 cells.

**Conclusions:**

Our study suggests that EphA2 signaling up-regulates the expression of the TJ-protein claudin-2 that plays an important role in promoting cell proliferation and tumor growth in NSCLC cells. We conclude that receptor EphA2 activation by ephrin-A1 induces tumor suppressor gene *cdx-2* expression which attenuates cell proliferation, tumor growth and thus may be a promising therapeutic target against NSCLC.

## Background

Tight junctions (TJ), the most apical cell-cell adhesion, owing to their cellular location are responsible for maintaining the cellular integrity. Any deregulation of the TJ characteristics could potentially lead to cellular transformation and acquisition of tumorogenesis potential [1]. However, emerging details from many studies related with claudin and cancer have implicated claudin family members in a wide range of human cancers. The expression of claudins would decrease during tumorogenesis as tight junctions are lost during cellular transformation, but it is understood that claudins are expressed in a tissue specific manner [2-11]. Down-regulation of claudins in cancer seems to be well understood, but increased expression of claudin contributing to neoplastic progression is less clear [1]. Aberrant tissue expression of certain claudins may contribute to neoplasia by directly altering TJ structure and function [1]. Furthermore it is also postulated that claudins may affect cell-signalling pathways [12].

*Cdx-2* is a transcriptional factor crucial to the normal proliferation and differentiation of intestinal epithelial cells [13], however little is known about the transcriptional program that controls genes involved in NSCLC tumor growth. In colorectal cancer reduced expression of *cdx-2* has been reported in rodents and humans [14,15]. In addition, *cdx-2* null mice embryos failed to survive and heterozygote’s developed intestinal tumors. Furthermore the polyps developed in the colon do not express *cdx-2* which indicates that loss of *cdx-2* promotes tumorogenesis [16]. *Cdx-2* regulates claudin-2 functions by binding to its 5’ flanking region and affects the expression of downstream pathway genes [17]. However, if receptor EphA2 activation with ephrin-A1 induced expression of *cdx-2* plays any role in NSCLC tumor growth is not known.

The Eph family of receptor tyrosine kinases plays key role in the development of cancer. The Eph receptors and ephrins were originally discovered as neuronal guidance and vasculature formation proteins during embryonic development [18]. Eph receptors and their ligands, ephrins are often dysregulated in malignant phenotypes including NSCLC [19-23]. However the precise role of these proteins in tumor growth is not well understood. Defining the role of EphA2 and ephrin-A1 in NSCLC is particularly important, as EphA2 receptor is highly expressed in NSCLC which contributes to tumor development. The aim of our study was to investigate the underlying mechanisms of tumor suppressor effect of ephrin-A1 in NSCLC. We report a novel mechanism of ephrin-A1 mediated attenuation of NSCLC tumor growth due to down regulation of claudin-2 and induction of tumor suppressor gene *cdx-2*. Thus providing the evidence that receptor EphA2 may be a promising therapeutic target for NSCLC.

## Methods

### NSCLC cell culture

A549 NSCLC cell line was obtained from American Type Culture Collection (Manassas, VA) and NCI-H2126, NCI-H838, NCI-H522, NCI-H23 NSCLC cell-lines were a kind gift from Dr. Frederic Kaye, MD, Division of Haemato-logy/Oncology, University of Florida, Gainesville, Florida. The NSCLC cells were resuspended in RPMI-1640 (Gibco Laboratories, Grand Island, NY) containing 10% FBS, penicillin (100 U/ml) and streptomycin (100 μg/ml). The cells were plated in 100 mm Petri dishes (Corning Costar Corporation, MA) and incubated at 37°C in 5% CO_2_ and 95% air. The cell culture medium was changed on alternate days. When the cells were confluent they were trypsinized and seeded into 100 mm culture dishes or transwell chambers as required for different assays.

### Construction of vectors containing ephrin-A1 (EFN-A1) and EphA2 and transient transfection of NSCLC cells

The gene transfer vector, pcDNA3.2/V5-DEST was used as an expression vector for the expression of ephrin-A1 (EFN-A1), and receptor EphA2, and pcDNA3.2/V5/CAT was used as a control vector (Invitrogen, Carlsbad, CA) as reported earlier [24]. For the over expression of *cdx-2* gene, pcMV6-XL5 was used as an expression vector for *cdx-2* and control vector in A549 cells (Origene Technologies, Inc.; Rockville, MD). The cloned vectors were designated as pcDNA-EFN-A1, pcDNA-EphA2 and pcMV-*cdx2* respectively. The control vectors were designated as Empty vector or pcMV-control. The NSCLC cells were transfected with vectors using lipofectamine-2000 reagent (Invitrogen, Carlsbad, CA). The transfected cells were used for further experiments.

### Transfection of NSCLC cells

The siRNA targeting the receptor EphA2 and *cdx-2* were designed using Oligoperfect design (Invitrogen, Carlsbad, CA). A549 cells were plated into 6-well plates or 35 mm plates as required for the experiments. The cells were allowed to adhere for 24 hours. The transfection of siRNA was performed using lipofectamine-2000 (Invitrogen) according to the manufacturer’s recommendation. The concentration of siRNA used was 100nM. After 4 hours of transfection, the culture medium with serum was added. The assays were carried out 48 hours post-transfection as reported earlier [25].

### Total RNA isolation and quantitative real time PCR analysis

Total RNA from cultured NSCLC cells was isolated and diluted with RNase-free water to 100 ng/mL; then, 10 μl of each sample were reverse transcribed into complementary DNA (cDNA) as reported earlier [22]. In brief, after the reverse transcription reaction, 80 μl of RNase free water were added to each sample. Ten microliters of diluted cDNA product were mixed with 25 μl of SYBR Green JumpStart Taq ReadyMix, 0.5 μl of internal reference dye, and 2.0 μl of specific oligonucleotide primers to a total volume of 50 μl for quantification of the real time polymerase chain reaction (PCR) (Table 1). The quantification of real-time PCR was performed by using the SYBR Green method on the Applied Biosystems 7500 Real Time PCR System with the following profile: 1 cycle at 94°C for 2 minutes; 40 cycles at 94°C for 15 seconds, at 60°C for 1 minute, and at 72°C for 1 minute for the elongation step. The real-time PCR products were confirmed by electrophoresis on 2% agarose gels. Data analysis was carried out by using the ABI sequence-detection software using relative quantification. The threshold cycle (Ct), which was defined as the cycle at which PCR amplification reaches a significant value was expressed as the mean value. The relative expression of messenger RNA (mRNA) was calculated by using the ΔCt method (where ΔCt was the value obtained by subtracting the Ct value of the housekeeping gene β-actin mRNA from the Ct value of the target mRNA). The amount of the target relative to β-actin mRNA was expressed as 2 ^-(ΔCt)^.

**Table 1:** Primers Used in Quantitative Reverse Transcriptase-Polymerase Chain Reaction analysis

**Gene Name**	**Forward Primer (5'-3')**	**Reverse Primer (5'-3')**
Claudin-2	GACCCCTAAGGCTGAGGAAC	AGAAGAGGAGGCCCAAGGAAG
*Cdx-2*	GCCAGGTCCTCTGAGAAGTG	CCTCTGAGAGCCAGGTCTGT
*Cdx-2*-siRNA	GACAAGGACGUGAGCAUGUACCCUA	UAGGGUACAUGCUCACGUCCUUGUC
18srRNA	AAACGGCTACCACATCCAAG	TAACGAGGATCCATTGGAGG

### Western blot analysis

NSCLC cells were cultured in 60 mm size cell culture dishes (Fisher Scientific, Pittsburgh, VA) to confluence and the cells were lysed in lysis buffer with the method reported earlier [26]. Protein was estimated by BCA method (PIERCE, Rockford, IL) and equal amount of protein (20 μg/lane) were loaded. Proteins in the sample were separated in denaturing sodium dodecyl sulphate (SDS) polyacrylamide gels (Bio-Rad), and transferred electrophoretically onto polyvinylidene difluoride membrane (Immobilon-P, Millipore, Bedford, MA). The blots were blocked with 5% Blotting Grade Blocker Non-fat Dry milk (Bio-Rad, Hercules, CA) for 1 hr on shaker at room temperature, and were overnight incubated at 4°C with respective antibodies - rabbit EphA2 antibody, rabbit *cdx-2* antibody (Cell signaling, Beverly, CA) and rabbit claudin-2 antibody (Invitrogen, Grand Island, NY), at 1:1000 dilutions. After washing, they were incubated with the secondary antibody (horseradish peroxidase-conjugated goat anti-rabbit IgG Ab) at a dilution of 1:1000 for 1 hr. Finally respective proteins were detected by enhanced chemiluminescence (ECL, Amersham Pharmacia Biotech). The Molecular mass (kDa) of the proteins was determined using the prestained protein marker (Bio-Rad).

### NSCLC cell proliferation

NSCLC cell proliferation was assessed by using an assay based on cleavage of the tetrazolium salt WST-1 to formazan by cellular mitochondrial dehydrogenases (Roche, Indianapolis, IN) as reported earlier [22]. With this assay, an increase in the number of viable cells results in an increase in the overall activity of the mitochondrial dehydrogenases in the sample. The augmentation in enzyme activity leads to an increase in the formazan dye formed. The formazan dye formed was quantified by using a plate reader at 450 nm. A549 cells were plated in 96-well microplate at a density of 0.5 X 10^5^ cells per well. The cells were transfected with pcDNA-EFNA1, pcDNA-EphA2, siRNA for EphA2 or with scrambled siRNA (sc-siRNA), *cdx-2*-siRNA by using lipofectamine-2000 reagent, and a few wells were left untreated. The negative controls received serum-free media, and some of the wells were activated with recombinant ephrin-A1. Cells were allowed to incubate for 48 hours. Then, the WST-1 reagent was applied for 4 hours to measure cell proliferation. The cell proliferation was assessed in triplicate. The data are presented as a percentage of negative control proliferation with *P* values *<0.05* were considered significant.

### Immunofluorescence microscopy

Claudin-2 expression in NSCLC cells was analyzed by using confocal laser-scanning microscopy (Zeiss LSM 510, Axiovert 100 M; Zeiss, Thornwood, NY), as reported previously [22]. In brief, the cells were cultured to confluence on gelatinized glass cover slips and fixed in 5% paraformaldehyde (with 50 mM phosphate buffer) in 50% Tris wash buffer (TWB). The glass cover slips were rinsed 3 times and permeabilized with 1.2% Triton X-100 for 5 minutes, rinsed 3 times, incubated with 1% bovine serum albumin (BSA) in 100% TWB for 1 hour, then stained for the expression claudin-2 using primary antibody rabbit anti-claudin-2 at 1:150 dilution and secondary antibody goat anti-rabbit immunoglobulin G conjugated with fluorescein isothiocyanate (FITC) (Zymed Laboratories, San Francisco, CA). 4, 6-diamino-2-phenylindole (DAPI) was used as a nuclear stain.

### NSCLC tumor growth in matrigel

A 48-well culture plate was coated with 200 μl of matrigel per well and then allowed to polymerize for about 1–2 hours at 37°C. NSCLC cells at a density of ~1 x 10^3^ cells per well were plated in 0.3 ml of 2% FBS containing RPMI-1640 as reported earlier [24]. The cells were activated with ephrin-A1 or transfected with plasmid pcDNA-EphA2 or pcDNA-EFNA1 or siRNA for EphA2 or *cdx-2* or empty vector by using lipofectamine-2000 and few wells were left untreated as controls, media were changed every three days. The number of colonies formed was recorded after 7 days of incubation. 4–6 randomly chosen fields (x10) from the sample were photographed.

### Statistical analysis

The Sigma Stat 3.5 statistical software program was used to calculate statistical significance. Kruskal-Wallis One Way Analysis of Variance (ANOVA) was used to compare the experimental groups from the control groups. The post hoc test Holm-Sidak method was applied for pairwise comparisons. The differences at *p <* 0.05 were considered statistically significant.

## Results

### NSCLC cells express receptor EphA2 and Caludin-2

**Figure 1:**
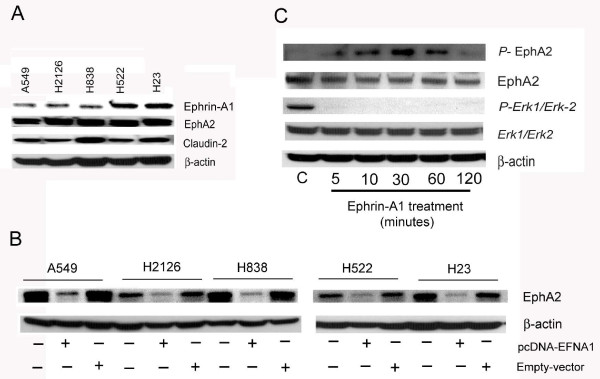
**NSCLC cells express receptor EphA2, Ephrin-A1 and Caludin-2.** Plate **A**: NSCLC cell lines (A549, H2126, H838, H522, H23) express receptor EphA2, ephrin-A1, and claudin-2**.** Expression of EphA2, ephrin-A1, and claudin-2 was analyzed by Western blot analysis. Plate **B**: Western blot analysis of EphA2 protein expression the β-actin was probed to demonstrate equal sample loading. NSCLC cell lines were either transfected with pcDNA-EFNA1 (pcDNA-EFNA1 is vector containing ephrin-A1 construct) or empty vector and receptor EphA2 expression was analyzed. Plate **C**: A549 cells were activated for 5 minutes to 120 minutes with ephrin-A1. Western blot analysis of phosphorylated EphA2 and *Erk1/Erk2* was performed, the β-actin was probed to demonstrate equal sample loading. Data presented is the representative of three separate experiments.

Proliferating NSCLC cells (A549, H2126, H838, H522, and H23) expressed receptor EphA2, and claudin-2 and the expression of ephrinA1 was variable (Figure 1A). Receptor EphA2 was over expressed in all the five NSCLC cell lines tested. However highest expression of claudin-2 was noticed in A549, H838 and H23 cell lines when compared to H2126 and H522 cell lines. Whereas, the expression of ephrin-A1 was dysregulated. A549, H2126 and H838 cells showed decreased expression of ephrin-A1 when compared to H522 and H23 (Figure 1A). In addition NSCLC cells were transiently transfected with vector containing ephrin-A1 construct (pcDNA-EFNA1) or empty vector and the expression of receptor EphA2 was determined in all NSCLC cell lines selected. Ephrin-A1 activation down regulated the expression of EphA2 when compared to empty vector transfected NSCLC cells (Figure 1B). In addition, A549 cells were also activated with recombinant ephrin-A1 for varying time (5, 10, 30, 60 and 120 minutes) and the activation/phosphorylation of receptor EphA2 was evaluated. Ephrin-A1 activation induced phosphorylation of receptor EphA2 up to 60 minutes in A549 cells. However, at 120 minutes the phosphorylation was down regulated. The total receptor expression was comparatively decreased when compared to control at 120 minutes (Figure 1C). The EphA2 signaling pathway activation was also determined by evaluating *Erk1/Erk2* activation in NSCLC. EphrinA1 activation inhibited the phosphorylation of *Erk1/Erk2* MAPK in NSCLC when compared to control cells (Figure 1C).

### Ephrin-A1 down regulated claudin-2 expression in NSCLC cells

**Figure 2:**
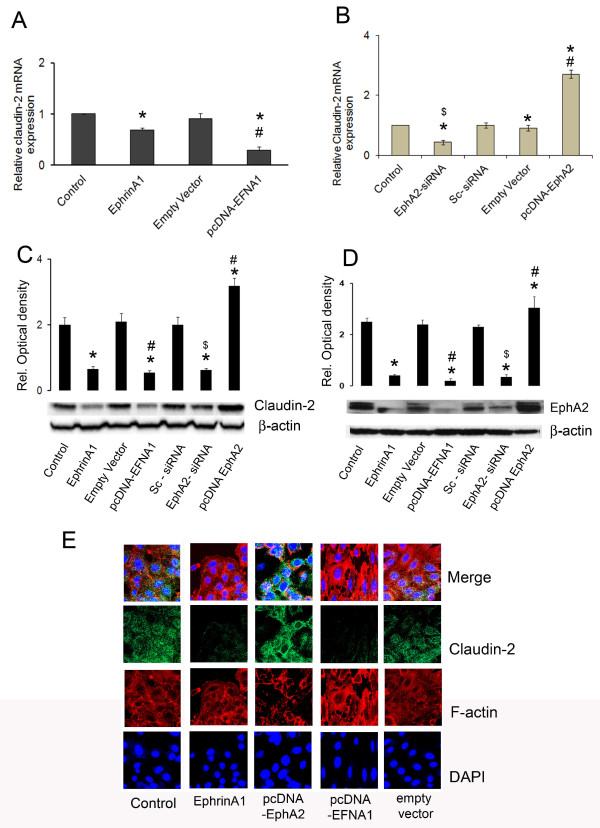
**Ephrin-A1 activation decreases claudin-2 expression in NSCLC cells.** Relative expression of claudin-2 mRNA normalized to endogenous control. Plate **A**: Cells were either activated with ephrin-A1 or transfected with pcDNA-EFNA1 (vector containing Ephrin-A1 construct) or empty vector. Plate **B**: Cells either silenced with EphA2 siRNA or transfected with pcDNA-EphA2 (pcDNA-EphA2 is vector containing EphA2 construct) Plate **C**: Claudin-2 protein expression by Western blot analysis, β-actin was probed to demonstrate equal sample loading. Plate **D**: Western blot analysis of EphA2 protein expression the β-actin was probed to demonstrate equal sample loading. Data presented was the mean ± SEM of three independent experiments, *p < 0.001 compared to control; *#p < 0.001* compared to empty vector; *$p < 0.001* compared to sc-siRNA. Plate E: Claudin-2 expression in NSCLC as observed by immunofluorescence analysis. The photomicrographs show immunofluorescence staining of claudin-2 in NSCLC. Blue colour represents the nuclear stain DAPI (4,6-diamino-2-phenylindole), Red colour represents Rhodamin phalloidin for F-actin filament stain, and green colour represents Alexa Flour 488 labelled for claudin-2 expression. The data presented is a single representative of three similar observations Scale Bar = 40 μm.

Ephrin-A1 activation of receptor EphA2 down regulated the expression of claudin-2 in NSCLC cells. To identify whether decrease in claudin-2 expression is directly due to decrease in EphA2 receptor expression, A549 cells were transfected with pcDNA-EFNA1 or empty vector or left untransfected. In comparison to empty vector the transient transfection of A549 cells with pcDNA-EFNA1 resulted in significantly decreased claudin-2 expression (Figure 2A). This suggested that activation of receptor EphA2 with the ligand ephrin-A1 induced rapid reduction of claudin-2 expression that plays a major role in maintaining cellular integrity. In addition the expression of claudin-2 and receptor EphA2 was also evaluated by Western blot analysis. The decreased expression of receptor EphA2 further confirmed that treatment of A549 cell with ephrin-A1 down regulates the expression of receptor EphA2 and claudin-2 (Figure 2C, -2D). These data suggests that ephrin-A1 treatment inhibits the expression of oncogenic protein EphA2 and claudin-2 in NSCLC cells.

In order to determine if over expression of the receptor EphA2 in A549 cells promotes claudin-2 expression, A549 cells were transiently transfected with pcDNA-EphA2 and the expression of claudin-2 was evaluated. A significant increase in the expression of claudin-2 was noted when compared to empty vector transfected cells (Figure 2B and C). To investigate whether this increase in claudin-2 expression was directly due to the increases in EphA2 receptor expression, NSCLC cells were transfected with EphA2-siRNA. In A549 cells, silencing the EphA2 receptor with siRNA significantly reduced the claudin-2 expression when compared to sc-siRNA transfected cells (Figure 2B and C). These results suggest that increased EphA2 expression modulated claudin-2 expression, which may play an important role in tumor growth in NSCLC.

### Claudin-2 expression decreased in Ephrin-A1 treated A549 cells

In order to evaluate the cellular distribution of caludin-2, and morphological changes in activated cells, A549 cells were activated with ephrin-A1 and analyzed by immunofluorescence microscopy. Fluorescence immuno-staining analysis revealed that A549 cells showed punctuated expression of claudin-2 whereas, ephrin-A1 activation decreased claudin-2 expression (Figure 2E). In cells transfected with pcDNA-EFNA1 a marked decrease in claudin-2 expression was noticed. In addition, ephrin-A1 activated cells showed distorted cytoskeleton, and rounded morphology. However, in cells transfected with pcDNA-EphA2 a dense and higher expression of claudin-2 was noticed when compared to control and empty vector transfected cells. These data suggests that over expression of receptor EphA2 promotes the expression of claudin-2 in NSCLC cells. Ephrin-A1 activation or transfection of cells with plasmid containing the ephrin-A1 construct inhibits the expression of claudin-2 confirming its anti-tumor effects on NSCLC cells.

### Ephrin-A1 treatment increased *cdx-2* expression in NSCLC cells

**Figure 3:**
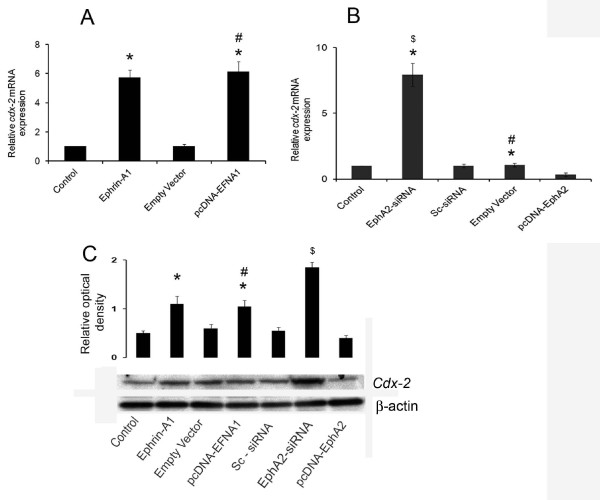
**Transfection of pcDNA-EFNA1 increased whereas transfection of pcDNA-EphA2 attenuated*cdx-2 *expression in NSCLC cells.** Plate **A**: cells were either activated with ephrin-A1 or transfected with pcDNA-EFNA1 (pcDNA-EFNA1 is vector containing ephrin-A1 construct). *Cdx-2* mRNA expression was analyzed by quantitative real time PCR. Plate **B**: Cells either silenced with EphA2 siRNA or transfected with pcDNA-EphA2 (pcDNA-EphA2 is vector containing EphA2 construct) Plate **C**: Western blot analysis of claudin-2 protein expression, the β-actin was probed to demonstrate equal sample loading. The Western blot represents three similar observations. Data presented is the mean ± SEM of three independent experiments. *p < 0.001 compared to control, *#p < 0.001* compared to Empty vector, *$p < 0.001* compared to sc-siRNA.

*Cdx-2* a tumor suppressor gene, found to be tissue specific in its expression. Ephrin-A1 activation induced more than five folds increases in *cdx-2* mRNA expression in NSCLC cells (Figure 3A). In addition this expression was also confirmed in the cells transfected with plasmid containing ephrin-A1 construct pcDNA-EFNA1. The transfection with pcDNA-EFNA1 also showed a significant increase of *cdx-2* expression in A549 cells when compared to empty vector (Figure 3A). Furthermore, the cells were also transfected with plasmid pcDNA-EphA2 or empty vector and the expression of *cdx-2* was evaluated. A remarkable reduction in the expression of *cdx-2* was noticed in cells transfected with pcDNA-EphA2. However, knockdown of EphA2 gene with siRNA-EphA2 significantly upregulated the expression of *cdx-2* in A549 cells when compared to sc-siRNA transfected cells (Figure 3B). These results suggest that unregulated/lost expression of *cdx-2*, a tumor suppressor gene may result to increased EphA2 expression in A549 cells. Furthermore *cdx-2* expression with activation of ephrin-A1 or transfection with pcDNA-EFNA1 or PcDNA-EphA2 in A549 cells was also confirmed with Western blot analysis (Figure 3C). These data suggests that activation of receptor with ephrin-A1 up regulates the expression of tumor suppressor gene *cdx-2*. Silencing the expression of EphA2 receptor also increases the expression of *cdx-2* which indicates that knockdown of EphA2 either by ephrin-A1 activation or by silencing interference RNA could be potential in inhibiting the oncogenic effect of receptor EphA2 and tumor growth.

### Silencing *cdx-2* expression blocked ephrin-A1 mediated inhibition of claudin-2 expression in NSCLC cells

**Figure 4:**
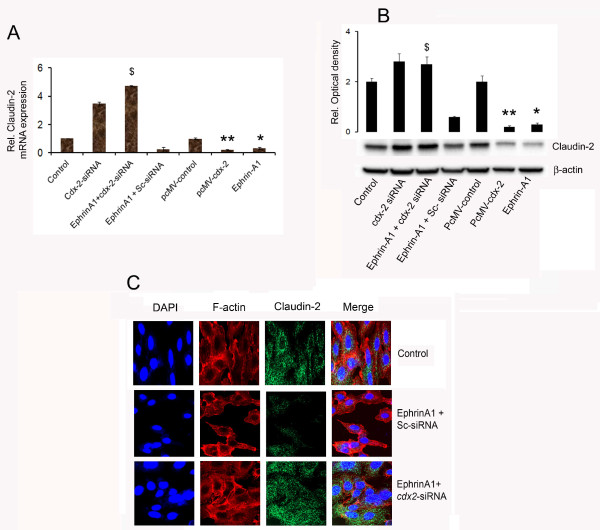
**Silencing *cdx-2 *expression restores Ephrin-A1 mediated inhibition of Claudin-2 expression in NSCLC cells.** Plate **A**: Relative mRNA expression of claudin-2 normalized to endogenous control. (pcDNA-EFNA1 is vector containing ephrin-A1 construct). Plate **B**: Claudin-2 expression was analyzed by Western blot, data represents mean ± SEM of three independent experiments. *p < 0.001 compared to control, *# p < 0.001* compared to Empty vector, *$p < 0.001* compared to *cdx2*-siRNA alone. **p < 0.001 compared to control. Plate **C**: Immunofluorescence analysis of claudin-2 expression in NSCLC. Blue colour represents the nuclear stain DAPI (4, 6-diamino-2-phenylindole), Red colour represents Rhodamin phalloidin for F-actin filaments, and green colour represents Alexa Flour-488 labelled for claudin-2 expression. The data presented is a single representative of three similar observations. Scale Bar = 40 μm.

Activation of A549 cells with ephrin-A1 or transfection with pcDNA-EFNA1 resulted in significant decrease of claudin-2 expression. In addition over expression of *cdx2* by using pcMV-*cdx-2*, a plasmid with *cdx2* construct resulted in significant decrease of claudin-2 expression (Figure 4A and B). Moreover, when A549 cells were transfected with *cdx-2-* siRNA and activated with ephrinA1 the expression of claudin-2 expression was significantly upregulated (Figure 4A). Furthermore Immunofluorescence staining for the expression of claudin-2 also confirmed that silencing *cdx-2* gene and activation with ephrin-A1 resulted in enhanced expression of claudin-2 (Figure 4C). Our results suggest that EphA2 signaling promoted claudin-2 expression in A549 cells and downregulated the expression of tumor suppressor gene *cdx-2*. These data also suggests that ephrin-A1 mediated down regulation of claudin-2 expression in A549 cells is dependent on *cdx-2.*

### Silencing *cdx-*2 expression blocked Ephrin-A1 mediated inhibition of cell proliferation in NSCLC cells

**Figure 5:**
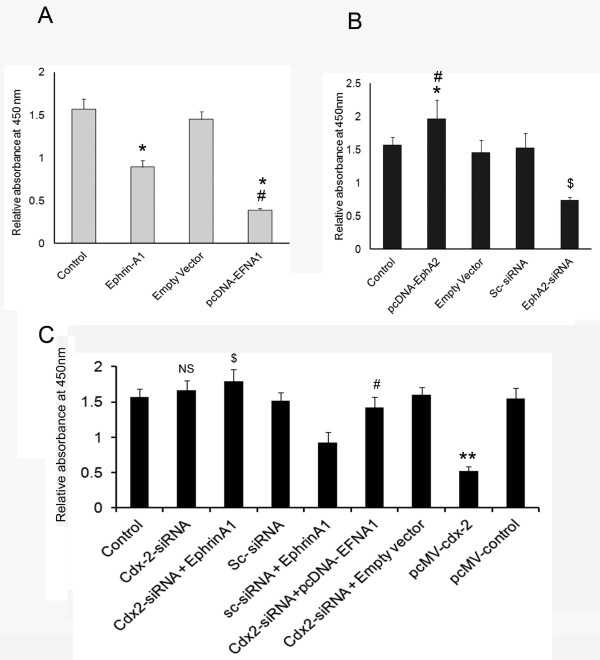
**Ephrin-A1 activation attenuates NSCLC cell proliferation.** Proliferation of NSCLC cells as measured by WST-1 assay. Plate **A**: Proliferation in cells either activated with ephrin-A1 or transfected with pcDNA-EFNA1 (pcDNA-EFNA1 is vector containing ephrin-A1 construct). Plate **B**: Proliferation in cells either silenced with EphA2 siRNA or transfected with pcDNA-EphA2 (pcDNA-EphA2 is vector containing EphA2 construct). Plate **C**: Proliferation in *cdx-2* siRNA transfected cells that were treated with ephrin-A1 ligand or pcDNA-EFNA1 transfected. Data presented is the mean ± SEM of three independent experiments. *p < 0.05 compared to control; $ *p < 0.05* compared to ephrin-A1 orpcDNA-EFNA1 alone, # *p < 0.001* compared to empty vector or sc-siRNA + ephrinA1, NS is not significant.

Ephrin-A1 activation and transfection with ephrin-A1 vector pcDNA-EFNA1 resulted in suppression of proliferation in A549 cells when compared to control or empty vector transfected cell (Figure 5A). Silencing of EphA2 receptor by EphA2-siRNA also showed decreased cell proliferation as compared to sc-siRNA or control (Figure 5B). In addition, the cells were transfected with pcDNA-EphA2 that showed a significant increase in proliferation when compared to empty vector or control cells. However, when cells were transfected with *cdx2*-siRNA and subsequently activated with ephrin-A1 the proliferation of cells was restored and showed close to control cell proliferation rate. Moreover, knockdown of *cdx-2* with siRNA and transient transfection with pcDNA-EFNA1, resulted in significant increase in cell proliferation compared to pcDNA-EFNA1 transfected cells (Figure 5A, -5C). The transfection of *cdx-2*-siRNA alone showed slight increase in the proliferation rate but was not significant when compared with control. These results suggest that ephrin-A1 induced increased expression of *cdx-2* suppressed proliferation of NSCLC. The transfection of NSCLC with *cdx2-*siRNA followed by ephrin-A1 activation blunted ephrin-A1 mediated inhibition of A549 cell proliferation.

### Ephrin-A1 activation inhibited tumor growth and silencing *cdx-2* expression blunted Ephrin-A1 mediated suppression of tumor growth in NSCLC cells *in vitro*

**Figure 6:**
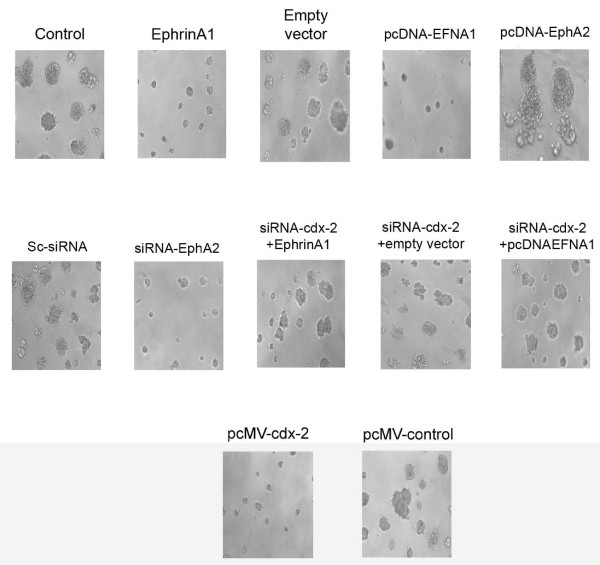
**Ephrin-A1 ligand activation decreases tumor growth in NSCLC.** Equal number of A549 cells was seeded in matrigels after various treatments as described and after 7 days of culture tumor growth was recorded by a SPOT digital camera attached to Nikon microscope. The data presented are a single representative of three similar but independent experiments. Magnification = 5 μM.

A549 cells were activated with eprhin-A1 or transfected with a plasmid pcDNA-EFNA1, or empty vector, or left untransfected as control. The transfected or control cells were plated on 3-D matrigels to determine the tumor growth *in vitro*. The tumor growth was studied for 2 weeks. Microscopic examination revealed that A549 cells activated with ephrin-A1 and those transfected with pcDNA-EFNA1 showed suppressed tumor growth in matrigel as compared to empty vector transfected or control cells (Figure 6). In contrast NSCLC cells transfected with pcDNA-EphA2 showed aggressive tumor growth which was significantly larger in size as compared to tumors grown after silencing the EphA2 with siRNA. In addition, when the A549 cells were transfected with *cdx-2* siRNA and subsequently treated with the ephrin-A1 or transfected with pcDNA-EFNA1 we noticed significantly increased tumor formation as compared to cells either activated with ephrin-A1 alone and pcDNA-EFNA1 transfected cells (Figure 6). Taken together these results indicate that when A549 cells transfected with pcDNA-EphA2 to over expressed receptor EphA2 increased claudin-2 expression was observed which promoted the A549 cell proliferation. Activation of NSCLC cells with ephrin-A1 leads to tumor growth inhibition via *cdx-*2 expression.

## Discussion

The major finding of our present study is that receptor EphA2 is over expressed in NSCLC cell lines which promotes tumor growth. In addition we also found that EphA2 promotes tight junction protein claudin-2 expression in A549 cells. However, the expression of ephrin-A1 was found to dysregulated and A549 cells showed minimal levels. There is accumulating evidence that activation of receptor EphA2 with its ligand ephrin-A1 attenuates tumorogenic potential of malignant cells [24,27,28]. The molecular mechanisms responsible for tumor suppressive effects of ephrin-A1 are still elusive. In the present study, we report that proliferating NSCLC cells showed enhanced expression of EphA2, and claudin-2. The activation of receptor EphA2 with ephrin-A1 inhibited the expression of EphA2 and claudin-2. To further examine the effect of ephrin-A1 on NSCLC we transfected the cells with vector expressing ephrinA1 construct, pcDNA-EFNA1. We found that forced expression of ephrin-A1 down regulated the receptor EphA2 and inhibited cell proliferation and tumor growth in 3D matrigel. In addition the activation of receptor EphA2 with ephrin-A1 induced phosphorylation of EphA2 and inhibited the downstream singling MAP kinase pathway *Erk1/Erk2*. Furthermore, the activation of EphA2 receptor with ephrinA1 induced *cdx-2*, a tumor suppressor gene in A549 cells. These data suggests that ephrin-A1 activation/transfection could effectively bind and activate endogenous EphA2 in NSCLC and led to internalization and degradation of EphA2. In order to understand if receptor EphA2 signaling modulates TJ protein claudin-2 we transfected the A549 cells with EphA2 expressing vector, pcDNA-EphA2. The expression of claudin-2 was higher in A549 cells transfected with pcDNA-EphA2 as compared to empty vector transfected cells or control cells. In addition, over expression of receptor EphA2 significantly enhanced tumor growth. Whereas silencing the expression of receptor EphA2 by siRNA, decreased the expression of claudin-2 and interestingly a significant up-regulation of *cdx-2* was noticed in NSCLC cells as compared to sc-siRNA transfected A549 cells. However, silencing *cdx-2* gene with siRNA and subsequent activation with ephrin-A1 or transfection with pcDNA-EFNA1 failed to inhibit tumor growth in A549 cells. Collectively these data suggests that an ephrin-A1 mediated anti-oncongenic effect is due to downregulation of EphA2, claudin-2 expression and induction of *cdx-2* gene in NSCLC.

EphA2 is an oncoprotein which promotes cell survival, abnormal cell growth and invasion in a number of malignancies, including NSCLC [18,20,21,29]. In malignant cells such as A549, due to dysregulated cell division and abnormal growth the cell-cell contacts are loose which hinders the interaction between neighbouring cells. The loss of contact among the adjacent cells results in accumulation of high levels of intracellular EphA2 and claudin-2 an integral component of tight junction. Tight junctions are the apical cell-cell adhesions that regulate paracellular permeability and are critical for cell polarity. Alteration in tight junction protein claudin-2, can cause the defects in normal regulation of growth factor receptor activation due to a differential distribution of the receptor and their respective ligands, which can be observed with respect to receptor EphA2 and its ligand Ephrin-A1 [30]. In this study, we attempted to understand the underlying mechanisms by which EphA2 over expression leads to enhanced or irregular claudin-2 expression via *cdx-2* modulation and promote tumor growth in NSCLC cells. Several studies reported that receptor EphA2 is over expressed in a number of malignancies [19,20,30]. Previously we have reported that EphA2 is over expressed in malignant mesothelioma cells (MMC) and posttranslational silencing of EphA2 significantly suppresses the proliferation and haptotactic migration of MMC [22,24]. In addition, EphA2 receptor activation in MMC by its ligand ephrin-A1 inhibited the *RAS* MAP kinase signaling pathways [23,24]. Our study in A549 cells revealed that receptor EphA2 signaling up regulates the TJ protein claudin-2. In turn, the over expression of claudin-2 along with EphA2 promotes A549 cell proliferation and tumor growth. It was reported that EGF signaling induced claudin-2 expression which promoted colonization of mammary tumor cells [31]. The up regulated levels of claudin-2 caused leaky cellular barriers in MDCK1 cells [32]. The junctional claudin-2 forms the selective cation channels that are sufficient to transform the functional “tight” junction into a “leaky” one [33]. The leaky barriers may contribute to increase uptake of nutrients and growth factor which promote exaggerated tumor colonization. In the present study we noted exaggerated tumor colonies formation when EphA2 was over expressed in A549 cells. It is plausible that over expression of receptor EphA2 promotes claudin-2 which in turn enhances tumor colonization of A549 cells.

We demonstrate that activation of receptor EphA2 with ephrin-A1 induced *cdx-2* expression and inhibited tumor formation. The over expression of *cdx-2* by vector pcMV-*cdx-2* resulted in downregulation of claudin-2 and attenuation of cell proliferation and tumor growth on matrigels. In addition silencing *cdx-2* expression using siRNA and activation with eprhin-A1 resulted in up regulation of claudin-2 in A549 cells. *Cdx-2, a* tumor suppressor gene is homeobox transcriptional factor that is known to control apical-basolateral polarity in mouse enterocytes and human colonic epithelial cells [34]. *Cdx-2* regulates epithelial cell polarity and morphogenesis through control of apical protein transport. At the transcriptional level, transcriptional factors such as *cdx-2* can bind to the promoter regions of various claudin genes and affect their expression [1,10]. In addition, certain characteristics of claudin-2 and *cdx-2* show similarity that both are critical for epithelial cell polarity [1,34]. Increased *cdx-2* expression was used as a marker for progression in gastric carcinogenesis [35], while some of the gastric cancers studies showed aberrant expression of *cdx-2* in intestinal metaplasia which is a subset of gastric adenocarcinoma [36]. The down-regulation of *cdx-2* mechanism was related to the induction of ulcer-associated cell lineage (UACL) [37]. In addition loss of *cdx-2* immunoreactivity was implicated as diagnostic feature in poorly differentiated colorectal adenocarcinoma [38]. Whereas, the reduced expression of *cdx-1* and *cdx-2* genes were associated with the development of enterocolitis in intestinal mucosa [39]. Furthermore, activation of *Ras* oncogene was associated with down regulation of the *cdx-2* in colon cancer cells [40]. All these studies confirm that expression of *cdx-2* gene though disease specific and tissue specific, the expression of *cdx2* was directly associated with tumor growth. The plausible mechanisms for the reduced *cdx-2* expression in carcinogenesis, could be that homeodomain proteins signifies roles in directing the cells to specified cell-phenotype during organogenesis in early stages of development. However, a reduction of *cdx-2* gene expression in the late stages such as in invasive tumors may be attributed to over expression oncogenic proteins which may lead to deviate from normal epithelial phenotype to the neoplastic phenotype [15].

It has been shown that, caudal-related homeobox gene cdx-2 is positively involved in the regulation of the human claudin-2 promoter activity [17]. The EphA2 signaling caused reduced expression of transcription factor *cdx-2* that hinder its binding to claudin promoter and thus cause irregular expression of claudin-2 which is reported to be increased in NSCLC cells in the present study. It is conceivable that due to the disrupted TJ or claudin-2 there is a disruption in epithelial cell polarity leading to leakage of large solutes passing across epithelial barriers to the other cells. Thus, the TJ disruption in premalignant neoplastic tissue can increase the proba-bility that it will develop into a complete carcinoma because of the continuous stimulation of cell division followed by disrupted natural barriers between growth factors and their receptors. The novel finding of our present study is that receptor EphA2 mediated the enhanced induction of functionally altered claudin-2 via down-regulation of tumor suppressor gene expression *cdx-2* in NSCLC cells. It is possible that activation of receptor EphA2 with ephrin-A1 downregulated claudin-2 and induced the expression of *cdx-2* suggesting oncogenic protein EphA2 play a major role in regulating *cdx-2* expression in NSCLC. Whereas, the forced expression of ephrin-A1 induced tumor suppressive signals via downregulation and degradation EphA2 and inhibited the oncogenic singling pathway in NSCLC. However, this needs to be further investigated.

## Conclusions

In conclusion, we present the first evidence that EphA2 signaling promotes the expression of claudin-2 in NSCLC cells. Activation of NSCLC with ligand ephrin-A1 suppressed the caludin-2 expression via the induction of transcriptional factor *cdx-2*. These studies suggest that targeting EphA2 by using ephrin-A1 may be a promising approach for the therapeutic inventions against NSCLC.

## Competing interests

The authors have no competing interests.

## Authors’ contributions

BSG, carried out the experiments. NN and BSG drafted the manuscript. NN and KAM critically analyzed, edited, reviewed and finalized the data. FK, for providing cell lines and editorial review, EPG provided the valuable comments and editorial review of the manuscript. All authors’ read and approved the final manuscript.

## Pre-publication history

The pre-publication history for this paper can be accessed here:

http://www.biomedcentral.com/1471-2407/12/309/prepub
